# The Association of Nursing Home Residents’ Pain with Preference‐Based Recreational Activity Attendance over time

**DOI:** 10.1002/alz.085303

**Published:** 2025-01-09

**Authors:** Kimberly S VanHaitsma, Allison R. Heid, Michael J Rovine, Karen Eshraghi, Nahida Akter, Katherine M Abbott

**Affiliations:** ^1^ The Pennsylvania State University, University Park, PA USA; ^2^ Independent Research Consultant, Philadelphia, PA USA; ^3^ University of Pennsylvania, Philadelphia, PA USA; ^4^ Abramson Senior Care, North Wales, PA USA; ^5^ Miami University’s Scripps Gerontology Center, Oxford, OH USA

## Abstract

**Background:**

Knowledge of nursing home (NH) residents’ everyday care preferences is foundational in that it allows for the delivery of person‐centered care and individualized care planning. However, little is known about how integrating preferences into care delivery impact outcomes of care. The Preference Match Tracker is an objective metric that tracks the number of recreation activities NH residents attend that match or is “congruent” with resident important preferences. Here, we explored how Preference Match Tracker data were linked to NH residents’ with and without dementia self‐reported pain over the course of one year.

**Method:**

The Preferences for Everyday Living Inventory (PELI) was used to assess important activity preferences for <I>N = </I>586 residents with varying levels of cognitive capacity from January 1, 2016, to March 13, 2020 (pre‐COVID‐19). Resident attendance at activities, as well as activities declined, per week was tracked as a metric for quality improvement. Pain data were collected from the MDS 3.0 and dichotomously coded as *pain* or *no pain*. We used PROC GLIMMIX in SAS 9.4, to model repeated measures logistic regression with time as the repeated measure, over 1 year. Covariates included age, gender, cognitive ability, length of stay, sensory impairment, and activities of daily living (ADL) functional ability.

**Results:**

There were no associations of age, gender, or hearing impairment with pain. Pain reduced over time. Greater cognitive capacity and shorter length of stay in the NH (< 90 days) were associated with more pain over time. More resident vision impairment and less ADL disability were also associated with less pain. After accounting for covariates, attending recreational activities was associated with less pain over time. Specifically, greater attendance at preferred activities was associated with less pain.

**Conclusion:**

Activity attendance may be beneficial for pain experience for NH residents with and without dementia, possibly by distracting residents from pain through meaningful engagement. Higher attendance, and specifically attendance in preferred activities, can support individuals experiencing pain, especially those new to a NH and in the early stages of dementia with greater cognitive capacity. Controlling pain may also promote residents’ capacity for engagement in activities, specifically preferred activities.

